# Tangled Physics: Knots Strain Intuitive Physical Reasoning

**DOI:** 10.1162/opmi_a_00159

**Published:** 2024-09-23

**Authors:** Sholei Croom, Chaz Firestone

**Affiliations:** Department of Psychological & Brain Sciences, Johns Hopkins University, Baltimore, MD, USA

**Keywords:** intuitive physics, visual perception, simulation

## Abstract

Whereas decades of research have cataloged striking errors in physical reasoning, a resurgence of interest in intuitive physics has revealed humans’ remarkable ability to successfully predict the unfolding of physical scenes. A leading interpretation intended to resolve these opposing results is that physical reasoning recruits a general-purpose mechanism that reliably models physical scenarios (explaining recent successes), but overly contrived tasks or impoverished and ecologically invalid stimuli can produce poor performance (accounting for earlier failures). But might there be tasks that persistently strain physical understanding, even in naturalistic contexts? Here, we explore this question by introducing a new intuitive physics task: evaluating the strength of knots and tangles. Knots are ubiquitous across cultures and time-periods, and evaluating them correctly often spells the difference between safety and peril. Despite this, 5 experiments show that observers fail to discern even very large differences in strength between knots. In a series of two-alternative forced-choice tasks, observers viewed a variety of simple “bends” (knots joining two pieces of thread) and decided which would require more force to undo. Though the strength of these knots is well-documented, observers’ judgments completely failed to reflect these distinctions, across naturalistic photographs (E1), idealized renderings (E2), dynamic videos (E3), and even when accompanied by schematic diagrams of the knots’ structures (E4). Moreover, these failures persisted despite accurate identification of the topological differences between the knots (E5); in other words, even when observers correctly perceived the underlying structure of the knot, they failed to correctly judge its strength. These results expose a blindspot in physical reasoning, placing new constraints on general-purpose theories of scene understanding.

## INTRODUCTION

**Figure 1.**
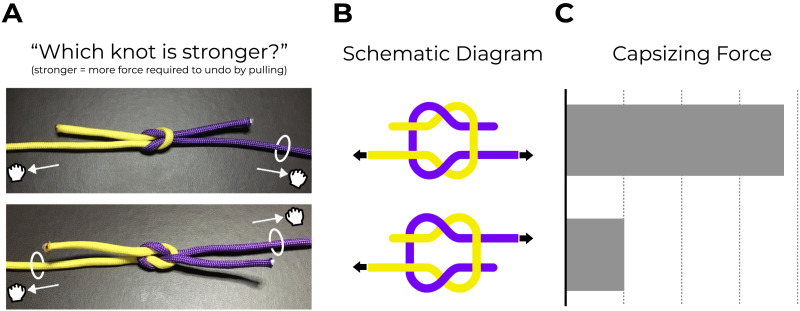
**(a)** Imagine pulling the longer ends of the two knots displayed here. Can you guess which one withstands the most pulling force? (The answer is revealed later in this caption). **(b)** Schematic diagram showing the topological organization of each knot from panel A. Notice, for example, the relative placement of the two pulled strands (i.e., those with arrows on them); in the top knot, the two pulled ends are on the same side as one another (yellow and purple both below), whereas in the bottom knot, the two pulled ends are opposite one another (yellow is below and purple is above). **(c)** Despite minimal topological differences, the reef knot (top) is substantially stronger than the grief knot (bottom), as measured by the force required for it to capsize (i.e., collapse or come apart). Readers can see this for themselves at https://perceptionresearch.org/knots, which features a video of author S.C. attempting to undo each of them.

Look at the images in Figure 1A. One of the knots depicted there is a staple of sailing and scouting practice, widely used across different cultures and historical eras to secure belongings, join lengths of string, and otherwise fasten and bind materials. The other is essentially a ‘trick’ knot; it is so insecure that it often comes apart on its own, and relying on it for anything practical would invite disastrous consequences (whether for your safety or the security of your belongings). Can you tell which is which? In other words, which knot seems like it would remain intact if pulled strongly at both ends, and which would easily capsize?

Judgments about physical scenarios and events pervade our daily lives, from deciding whether the stack of dishes in our sink can withstand another plate, to choosing how hard to push a child on a swing. However, the nature and accuracy of these judgments has been the subject of debate across different approaches and research traditions in psychology. Early work investigating physical reasoning cataloged many striking and surprising contexts in which physical intuitions sharply deviate from the principles of Newtonian physics. For example, when asked to predict the trajectory of an object dropped from an airplane, or to trace the path of a ball exiting a spiral tube, even highly educated college students (including those with formal physics education) make odd and persistent errors, such as believing that objects always fall straight down rather than maintaining their lateral momentum (Cook & Breedin, 1994; Gilden & Proffitt, 1994; McCloskey, 1983; McCloskey et al., 1980). These and other errors motivated theories of physical reasoning as a heterogeneous and inconsistent set of heuristics that are employed in specific contexts, with varying degrees of (in)accuracy (for a review, see Kubricht et al., 2017).

However, a different perspective has emerged more recently, driven by newer results that highlight surprisingly successful physical reasoning. For example, observers can correctly and rapidly predict whether and how a tower of blocks will fall (Battaglia et al., 2013; Firestone & Scholl, 2016, 2017), the relative masses of objects participating in collisions (Hamrick et al., 2016), and even the proportion of a poured liquid that will end up on either side of a partition (Bates et al., 2019). These and other successes have motivated a different account, according to which physical intuitions derive from a rich, probabilistic, generative model of the world and its physical laws, rather than the application of rough and ready heuristics. One especially intriguing hypothesis in this domain is that such models and simulations resemble the software architectures used in gaming environments (Battaglia et al., 2013; Ullman et al., 2017). According to this view, observers infer the future state of the world by running simulations in a mental “intuitive physics engine” (IPE), and treat the outputs of this engine (which may be subject to perceptual noise and uncertainty) as statistical samples from which to make physical inferences. These features of the IPE allow for sufficiently accurate predictions in most everyday scenarios (though they may also be subject to occasional illusions and biases, perhaps as a result of limited cognitive resources). More generally, accounts of this sort tend to embrace general-purpose approaches to physical reasoning, according to which the mind applies roughly the same principles and architecture to a wide variety of physical reasoning tasks.

### Reconciling Successes and Failure: Naturalism and Context

These two research traditions, one older and one more recent, offer conflicting perspectives on the nature and accuracy of intuitive physical reasoning. How do the more recent views emphasizing success account for the many observed failures in earlier studies?

A leading approach has been to explain away earlier failures by appealing to the contrived or impoverished nature of the stimuli and tasks used in previous studies. For example, whereas early work reported striking errors when subjects must use a pen to trace the future trajectory of a weight cut from a swinging pendulum (Caramazza et al., 1981), more recent work discovered that if the pendulum is animated and subjects must move a cup to catch the weight, they behave much more accurately (Smith et al., 2018). Indeed, many other intuitive misconceptions reported in early research may be ameliorated or abolished by the use of more naturalistic and dynamic stimuli and tasks, such as rich, animated scenes (Kaiser et al., 1992), more familiar and ecologically valid tasks and contexts (Kaiser et al., 1986), and measures that prompt simulated or imagined actions (Schwartz & Black, 1999); see also discussion in Fischer and Mahon (2022), who propose that “first-person” or user-oriented tasks produce better physical judgments than third-person problem solving. In light of these and other results, it has more recently been proposed that “the contrast between rich and calibrated versus poor and inaccurate patterns of physical reasoning exists as a result of using different systems of knowledge across tasks” (Smith et al., 2018), and that “when using more-realistic displays and actions, our intuitions actually closely match Newtonian dynamics” (Ullman et al., 2017).

Thus, intuitive physics research has expanded to include more familiar and ecologically valid physical reasoning tasks, and there is evidence that this addition of richness and context may account for some of the failures observed earlier. However, there are many physical systems and contexts that are part of our everyday lives but have remained almost completely unexplored in this literature. Might any of those domains put pressure on the above consensus? In other words, might there be a class of stimuli and tasks that both (a) are naturalistic, familiar, and intertwined with daily life, and yet (b) dramatically strain human physical scene understanding?

**Figure 2.**
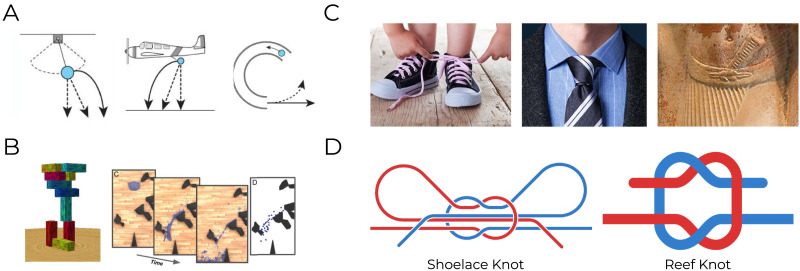
**Example stimuli from intuitive physics research.** **(a)** Early studies of intuitive physics revealed systematic errors in judgment. When participants are instructed to identify the trajectory of the blue target object, they are reliably inaccurate. For example, participants predict that a ball cut from a swinging pendulum or dropped from a moving plane will take a straight path to the ground rather than a curved one. Conversely, naive participants tend to believe that a ball exiting a spiral tube will continue on a curved trajectory rather than exiting on a straight path. (Adapted from Kubricht et al., 2017.) **(b)** More recent intuitive physics research has revealed more accurate and reliable judgments. When participants are instructed to judge the stability of a block tower or the flow of a poured liquid over obstacles, they demonstrate subtle and reliable understanding of these physical scenarios. This evidence has been taken to support a general-purpose mechanism for simulating the unfolding of physical scenes, especially when using naturalistic stimuli (as compared to earlier studies using diagrams). (Adapted from Bates et al., 2019; Hamrick et al., 2016.) **(c)** The present work explores intuitive judgments about knots. Knots are used in a wide variety of contexts, ranging from specialized activities such as sailing, rock climbing and survivalism to more mundane activities such as tying one’s shoelaces or a necktie. The rightmost image shows a reef knot (the same kind of knot seen in Figure 1A) around the belt of a figure in an Ancient Egyptian sculpture ca. 2350 BCE – evidence that these knots have been in use across cultures and time periods. **(d)** As shown in schematic diagrams, a typical shoelace knot is far more complex than the reef knot (and its variations) that we study here, and indeed even ‘contains’ a reef knot at its core.

Identifying such cases is important because it may reveal boundary conditions or constraints on the general-purpose nature of physical reasoning mechanisms. Discovering which stimuli and tasks are easy and which are difficult may serve as crucial data to ultimately inform a complete theory of physical scene understanding (since any such theory will have to account for both successes and failures).

### Introducing Knots to the Study of Physical Reasoning

Here, we introduce such a stimulus class to the study of intuitive physics, by exploring human judgments about *knots*. Knots are naturalistic stimuli that appear across cultures and time periods. For example, art from Ancient Egypt (ca. 2350 BC) depicts the classic “reef” knot around a person’s waist (Louvre, 1938), and there is similar evidence from Ancient Greece, Ancient and Imperial China, and even prehistoric societies that engaged in sewing and other clothwork (d’Errico et al., 2018; Leroi-Gourhan, 1982). It is often thought that knots predate human use of both fire and the wheel (Turner & van de Griend, 1996), and there is also evidence of cordage production among Neanderthals (Hardy et al., 2020); even non-human animals employ tangled structures in nest-building, predation, and other practices (for example, see Herzfeld & Lestel, 2005, for a fascinating ethnographic study of an orangutan who can tie “true” knots using her hands, feet, and mouth). Moreover, knots are widely used both in mundane scenarios (e.g., tying one’s shoelaces or the drawstring of a bag) and in more technical applications where one’s knot selection and skill can spell the difference between safety and peril (e.g., sailing or rock climbing). We’re also often tasked with *untying* knots, such as when headphone cords or necklaces become tangled in one’s pocket.

Knots can also be depicted in a variety of styles and representations, including naturalistic images and animations, as well as abstract idealizations and diagrams (both of the formats popular in previous intuitive physics research). Moreover, their physical properties can be precisely characterized. For example, recent research in the domains of topology and applied physics has simulated and experimentally investigated the physical mechanics of many popular knots (Patil et al., 2020), allowing for a ground-truth baseline against which to test human intuition. However, knots remain almost completely unexplored in intuitive physics research, despite suggestions that they may form a rich and promising domain for investigation (Santos et al., 2019).

**Figure 3.**
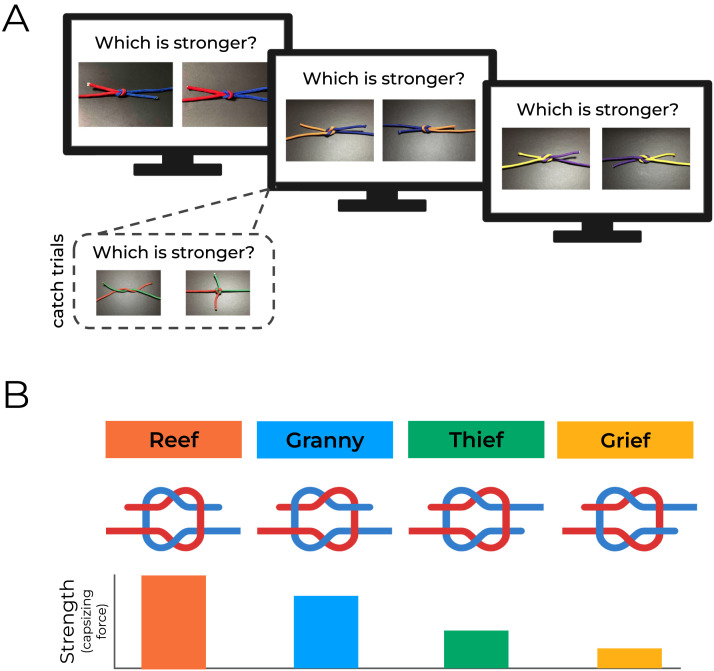
**Design and predictions of Experiment 1.** **(a)** Each monitor shows a sample trial of Experiment 1, which presents two knots on each trial. Participants simply answered which was stronger, using the criteria described in the main text and illustrated earlier in Figure 1. (Inset: Catch trials, which depicted a trivially easy strength contrast.) **(b)** Bar chart displaying the relative strengths of each knot in the RTGG knot series. If naive participants are sensitive to how the topological differences map onto differences in strength, then reef knots should be selected as the strongest in pairwise comparisons, grief knots least often, and so on for the other comparisons. Readers can experience this task for themselves at https://perceptionresearch.org/knots.

The present work enters this new domain by examining the ability of naive human subjects to evaluate the strength of various knots and tangles. As a case study, we focus on a series of 2-tangle knots that join lengths of string, known as the “reef”, “thief”, “granny” and “grief” series. These knots, depicted in Figure 3, are quite visually similar, and yet they vary widely in their stability, which is operationalized as the amount of force required for them to capsize. Reef knots (one of the most prevalent and recognizable knots in the world) are much stronger than thief knots; similarly, granny knots are much stronger than grief knots. This is true not only according to the cultural knowledge and practices of the communities that use (or avoid) these knots (such as sailors and scouts), but also according to recent scientific studies of them. For example, Patil et al. (2020) specifically examined the mechanics of this series of knots and concluded through computer simulations and real-world experiments that the received wisdom about these knots is accurately reflected in their physical behavior.

Surprisingly, the knots in this series are often distinguished only by the position of a single thread, and yet they differ dramatically in strength. In fact, the uppermost knot in Figure 1A (a reef knot) is many times stronger than the lowermost knot (a grief knot), despite their relatively minimal visual and topological differences. (Indeed, the Ashley Book of Knots, an authoritative and widely referenced source on knotcraft, calls the grief knot “hardly a practical knot” and instead considers it merely “an interesting trick”; Ashley, 1944).

Importantly, the knots mentioned here are (a) among the simplest knots that can be tied with two lengths of string, and (b) quite prevalent in daily life (even if they may not initially seem that way). For example, the standard “shoelace knot” that many of us tie every morning contains within it a reef knot (such that the reef knot is, by definition, *simpler* than the shoelace knot). Moreover, a granny knot is simply two half knots tied one after the other. Thus, chances are that *you* have frequently tied this knot without realizing it (e.g., to secure sweatpants or a bag, or simply in the course of tying your shoelaces; Skwarecki, 2023). Thus, if it turns out that ordinary people *cannot* easily intuit the strength of these simple and pervasive knots, then it is quite likely that even less familiar and/or more complicated knots (e.g., complex knots that take these knots as constituents, or entirely separate patterns of tangles) would be all the more challenging.

### The Present Experiments: Evaluating the Strength of Knots and Tangles

The tightly controlled nature of this group of knots, combined with the established hierarchy of their physical strength, makes them well suited to the present research question and easy to adapt to a psychophysical paradigm. Here, we present 5 experiments examining people’s intuitions about the physical dynamics of knots. Participants viewed images of these knots in various formats and presentation conditions (including photographs of the physical knots, digital renders from simulations, dynamic videos, and schematic diagrams) and were simply asked to evaluate their relative strengths under forced-choice conditions.

If performance on intuitive physics tasks derives from a general-purpose physical reasoning mechanism that approximates Newtonian physics (at least in naturalistic settings), then we might expect participants to reliably select the stronger knots, in line with their hierarchical organization. For example, reef knots should tend to be judged as stronger than the other three knots in the series, grief knots should be judged as weaker, and so on. However, if participants instead fail to appreciate these differences in knot strength (despite their naturalistic presentation and context), then this might reflect broader limits on physical reasoning. To foreshadow our key results: Across all experiments and presentations, participants failed to produce strength judgments consistent with Newtonian physics (Experiments 1–4), despite demonstrating accurate visual and topological understanding of the knots they were viewing (Experiment 5). Indeed, participants often gave *actively incorrect* rankings of the knot hierarchy within a given experiment (such that the findings do not merely reflect null results or chance performance). We suggest that these results put pressure on general-purpose accounts of physical scene understanding, and place new constraints on theories of how we reason about the physical world.

## EXPERIMENT 1: NATURALISTIC JUDGMENTS OF KNOT STRENGTH

Can naive human observers intuit the strength of visually similar but mechanically dissimilar knots? Experiment 1 investigated this question as described above, by evaluating whether observers could accurately judge the strength of reef, thief, granny, and grief knots. Since previous failures in physical reasoning have been attributed to contrived stimuli or a lack of context, we maximized naturalism in our stimuli by simply taking photographs of real knots tied with nylon rope. (Later experiments further enhance and probe both the naturalism and precision of this setup.)

### Method

#### Open Science Practices.

All data and materials supporting this experiment (and all others reported in this paper) are available at https://osf.io/xyq4h/. This study was not preregistered.

#### Participants.

50 participants were recruited online using Prolific and were compensated at an average rate of $10.50 per hour for their time. All participants were located in the United States. One participant was excluded from analysis due to failed attention checks (see below for more information).

#### Stimuli.

Stimuli consisted of photographs of the reef, thief, granny, and grief knots (hereafter RTGG), tied (by author S.C.) using 4 mm nylon rope. Each knot was tied in three separate colorways (red/green, yellow/purple, and orange/blue) and photographed from two different perspectives (front and back views of the knot), resulting in 24 total images. Each knot was roughly pulled taut, and tied to maximize visual similarity using the length of the bitter ends (the section of a rope that is tied off) as a reference. Each knot was photographed lying flat against a dark background and lit with neutral lighting. (In addition, two “catch” knots were created using a similar method; see below for more detail.)

#### Procedure.

Participants were told that their task was to evaluate knot strength, which was defined (and visually depicted) as being unlikely to come undone if you were to pull on the two long strands extending off-screen. (To ensure that these instructions were clear, participants had to pass a practice trial in which a very secure knot appeared next to loosely woven strings.) On each experimental trial, participants saw photographs of two knots at a time and were prompted to select the knot that appeared to be stronger by clicking on it. Feedback was not given. Since every trial only displayed two knots, each trial had either a correct or an incorrect answer, though some trials showed knots with greater strength differences than others. Participants saw every combination of the four knots possible, crossed with color and perspective (either the front or back of the knot), totaling 144 experimental trials. Additionally, four catch trials were dispersed at random through the task (these were the same images as the practice trials), and later used as exclusion criteria. Finally, subjects were also given a post-experiment survey in which they described any strategies they used to complete the task.

Readers can experience this task for themselves at https://perceptionresearch.org/knots.

### Results

One participant failed to answer all catch trials correctly, and so was excluded from further analysis, leaving 49 participants. (However, no result reported in this paper depends on these sorts of exclusions; in other words, all significant findings remain significant, in the same direction, even when no subjects are excluded at all.)

**Figure 4.**
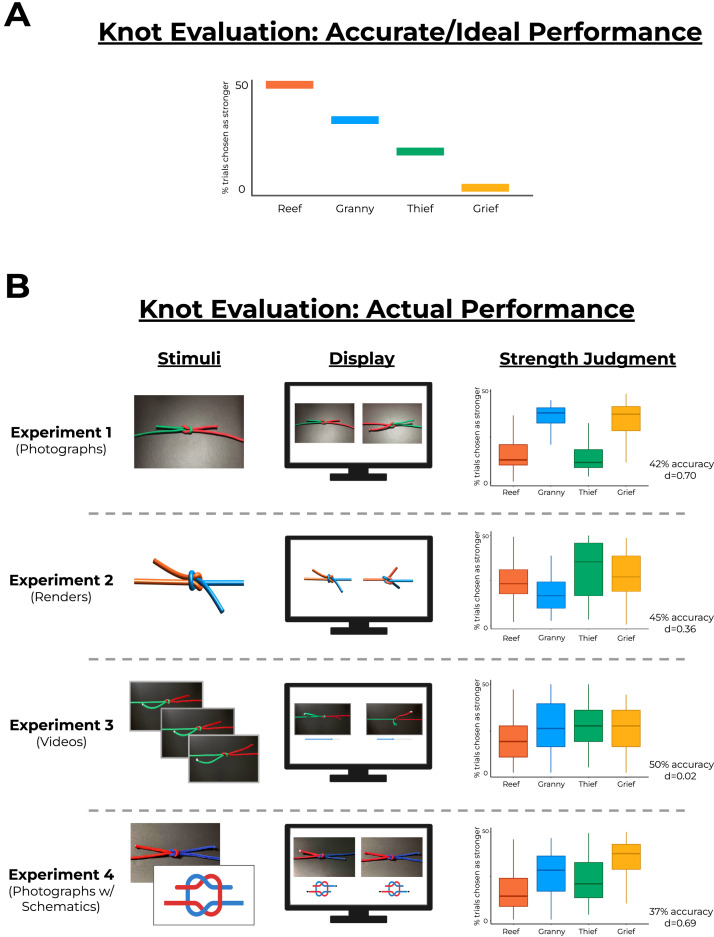
**Results of Experiments 1–4.** (**a**) ‘Accurate’ performance for the knot evaluation task. If subjects correctly represent knot strength (even subject to noise or error), the distribution of strength judgments should resemble the depicted ordering. Higher frequencies indicate that a knot won more pairwise comparisons throughout the experiment (i.e., was judged as stronger). (**b**) In fact, Experiments 1–4 show that participants fail to produce judgments consistent with ground-truth physics. Center line is the median, top and bottom of the boxes represent the interquartile range, and whiskers are minimum and maximum values excluding outliers. Importantly, responses were not merely random: As can be seen across experiments, responses were often quite consistent – just consistently *incorrect*. These results suggest that knots reliably strain physical reasoning.

We evaluate performance by examining how often a given knot is chosen relative to the others, across all trials. If intuitions about the relative stability of knots map on to their ground truth relative stability, then we should see a pattern that looks roughly like Figure 4A. Reef knots are the strongest of the four, so they should be selected the most often during the experiment, followed by granny, thief and finally grief knots, which are the weakest, and should rarely (if ever) be selected during the experiment.

However, as can be seen in Figure 4B, performance did not at all capture this hierarchy; in fact, performance was below chance. Participants selected the stronger knot on only 42.1% of trials (where chance is 50%; *t*(48) = 4.87, *p* < 0.001; *d* = 0.70), despite having demonstrated that they understood the instructions and correctly answered the catch trials. Breaking this performance down by knot type: Reef knots were chosen on 34% of the trials where they were shown (where chance is 50%), or on 17% of trials overall (where chance would be 25%). Granny knots were chosen 68% of the time (34% overall), Thief knots 32% (16%), and Grief knots 67% (33.3%). In other words, subjects showed little to no sensitivity to the large differences in strength between these visually similar knots.

To appreciate this pattern more precisely, consider how judgments of Reef knots (the strongest knots shown) compare to judgments of the other knots. First, Reef knots were chosen at almost identical rates as Thief knots, despite being quite different in strength. These two knots differ only in the placement of the bitter ends (Reef - same side; Thief - different side); even though this subtle difference has major consequences for knot strength, subjects evidently did not appreciate these consequences. Perhaps even more strikingly, however, Reef knots were consistently chosen as weaker than Granny and Grief knots, despite being substantially stronger than both of them. Indeed, Griefs (the weakest knot) were chosen 67% of the time they were shown (i.e., 33.3% overall), compared to Reefs (the strongest knot), which were chosen 34% of the time they were shown (i.e., 17% overall) – precisely the opposite of their actual relationship.

Moreover, using a computational approach developed for computing dominance hierarchies (e.g., the probability that competitor A beats competitor B, C, and so on) from a series of pairwise competitions (Fujii et al., 2014), we can calculate a knot rank hierarchy for each subject based on the outcomes of their pairwise strength judgments. Of the included subjects, the most popular rank order was *granny* > *grief* > *reef* > *thief* (33% of subjects), followed by *grief* > *granny* > *reef* > *thief* (27% of subjects), and then *granny* > *grief* > *thief* > *reef* (12% of subjects). Notably, none of these rankings is correct, nor even particularly close. Furthermore, not a single subject expressed the correct rank order.

Furthermore, this poor overall performance did not reflect random or unsystematic responding. To analyze the consistency of participants’ judgments, we assigned each participant and each knot pair a “consistency score”, corresponding to the proportion of trials where a participant picked the same knot in a given pairwise comparison. For example, on trials where participants saw a Reef and a Grief knot (24 trials total per participant), a participant who always answered Reef (i.e., 100% accuracy) received a consistency score of 1, and a participant who always answered Grief (i.e., 0% accuracy) also received a consistency score of 1. By contrast, a participant who answered Reef on 50% of Reef-Grief trials and Grief on 50% of Reef-Grief trials received a consistency score of 0 (with intermediate values calculated according to the formula *consistencyScore* = 2∣*proportionCorrect* − 0.5∣). This analysis revealed consistency scores well above 0 on all pairs, though consistency was much lower for Reef-Thief (mean consistency score = 0.22) and Granny-Grief pairs (mean consistency score = 0.23), which share most of their overall topology and differ only in the position of a single strand. Consistency was much higher for Reef-Granny (mean consistency score = 0.81), Reef-Grief (mean consistency score = 0.77), Granny-Thief (mean consistency score = 0.78), Thief-Grief (mean consistency score = 0.79). Thus, even though participants showed that they could discriminate between the knots (since they didn’t simply pick each knot with the same frequency) and understand what it means for a knot to be strong (since they passed the catch trials), they failed to grasp the relationship between the visual appearance of the knots and their strength. These results thereby provide initial evidence that knots strain physical reasoning.

## EXPERIMENTS 2–4: INCREASING PRECISION, RICHNESS AND NATURALISM

Experiment 1 provided initial evidence that knots pose a challenge to physical reasoning: When shown natural photographs of knots that vary greatly in strength, subjects failed to distinguish strong knots from weak ones. However, as with the classical physical reasoning errors reviewed earlier, it is possible that poor performance was driven by auxiliary factors that prevented subjects from accessing or demonstrating subtler and more accurate physical knowledge. For example: (1) Although the knots were hand-tied to maximize naturalism and ecological validity, this may have come at the cost of (inadvertent) inconsistencies across colorways, perspectives, and even knot type, which may have biased strength evaluations; (2) As static images taken from only one perspective (per image) and only two orientations (per knot), the stimuli may have lacked the full context that would be available when viewing a knot under real-world conditions (which permit dynamic sampling of different viewpoints, double-checking key perspectives and angles, etc.), in ways that may matter for engaging the operations of a mental physics engine; (3) It is unclear whether subjects could even recover the topological structures of the knots, perhaps due to one or more of the above-mentioned reasons, but perhaps due to the inherent difficulty of extracting topological organization from images.

Experiments 2–4 addressed each of these weaknesses directly. To ensure that the knots shown to subjects were accurate with respect to their physical properties, Experiment 2 used digital renders from software specifically designed to simulate knots under realistic physical conditions (including pulling force). To ensure that subjects could leverage dynamic information from many viewpoints, Experiment 3 presented subjects with scrollable videos of the knots rotating 360° in space. And to ensure that subjects had access to the underlying topology of each knot, Experiment 4 included schematic diagrams that make this topology explicit and unambiguous. If subjects continue to fail to appreciate knot strength even under these very accommodating conditions, this would be especially strong evidence that knots strain physical reasoning.

### Methods

All three experiments used a similar design to Experiment 1: A two-alternative forced-choice task between members of the RTGG series evaluated for strength. Each experiment recruited a new sample: Experiment 2 recruited 50 subjects to mirror Experiment 1, and Experiments 3 and 4 recruited 100 subjects each to increase statistical power. Of these, zero participants were excluded in Experiment 2 (for a total of 50 subjects), 16 subjects were excluded in Experiment 3 (for a total of 84 subjects) and 4 participants were excluded in Experiment for a total of (96 subjects). What differed primarily was the nature of the stimuli. Participants in each task were compensated at an average rate of $10.50 per hour for their time.

#### Stimuli.

Experiment 2 depicted the same knot series as Experiment 1, but digitally rendered in MATLAB using the procedure developed by Patil et al. (2020). The simulated knots had a 4 mm diameter, a bending modulus of 0.1 GPa, a Young’s modulus of 1 GPa, a Poisson’s ratio of 0.3, and 15 N of pulling force. The simulation was run to maximize visual similarity of the knots using the length of the bitter ends as a reference. Each knot was rendered against a transparent white background.

Experiment 3 used hand-tied knots like Experiment 1; but rather than photographs showing static images of the front and back of each knot, participants viewed interactive videos of each knot rotating 360°. All dynamic knot videos were recorded using an iPhone 11 and converted into a sequence of 126 frames each using kdenlive (https://kdenlive.org/). Each frame displayed a knot rotating along the z axis until it completed a full 360° rotation, working out to about 3° of rotation per frame. Participants could dynamically scroll through the video frames by dragging a scroll bar under each video. The frame displayed for each knot corresponded to the participant-initiated position of the scroll bar (i.e., if the scroll bar was in position 67, the 67th frame of the video would be shown). Participants could not advance to the next trial without at least partially scrolling through both videos.

Experiment 4 used the same static photographs from Experiment 1, but with the addition of schematic diagrams underneath each of the knot images. Each knot schematic was adapted from public domain images, and altered to match the colorways depicted in the knot photographs. Arrows were also added to the longer ends of each schematic to indicate the pulling direction participants should imagine when evaluating its strength.

### Results and Discussion

All three experiments failed to reveal accurate evaluations of knot strength, with performance at or below chance. (Note that the distinction between performing at chance vs. below chance is not crucial for our purposes; what matters most is that participants failed to perform *above* chance.)

In Experiment 2 (renders), overall performance was 44.8%, which was significantly different than chance, *t*(49) = 2.57, *p* < 0.05; *d* = 0.36. Despite similarly poor performance overall, the pattern differed from Experiment 1 with respect to the chosen hierarchy of knots. For example, while subjects in Experiment 1 clearly chose Granny and Grief knots more often than Reef and Thief knots, in Experiment 2 this pattern was more equivocal, though Thief and Grief knots were chosen marginally more often than Granny and Reef knots. Despite these differences, subjects were similarly consistent in their choices as in Experiment 1, with an average consistency score of 0.62 across all pairwise comparisons. Mean consistency scores for each pairwise comparison were as follows: Reef-Grief: 0.57; Reef-Granny: 0.50; Reef-Thief: 0.59; Granny-Grief: 0.55; Granny-Thief: 0.73; Thief-Granny: 0.67.

In Experiment 3 (videos), overall performance was 49.6%, which was not significantly different than chance, *t*(83) = 0.21, *p* = 0.83; *d* = 0.02. Consistency scores here averaged 0.55, with the following consistency scores for each pairwise comparison: Reef-Grief: 0.64; Reef-Granny: 0.62; Reef-Thief: 0.37; Granny-Grief: 0.52; Granny-Thief: 0.59; Thief-Granny: 0.66.

In Experiment 4 (schematics), performance was 36.9%, which was significantly lower than chance, *t*(95) = 6.76, *p* < 0.0001; *d* = 0.69. The pattern of results mirrors those of Experiment 1, with Grief knots and Granny knots being chosen as stronger more consistently than Reef and Thief knots, despite the diagrams unambiguously showing how the strands overlap. Participants showed an average consistency score of 0.65. Across pairwise comparisons, the mean consistency scores were as follows: Reef-Grief: 0.78; Reef-Granny: 0.70; Reef-Thief: 0.55; Granny-Grief: 0.52; Granny-Thief: 0.66; Thief-Granny: 0.72.

In other words, all of these variations not only failed to elicit accurate physical intuitions about knot strength, but in many cases also elicited *inaccurate* physical intuitions. (Full rank-order data for all subjects and all experiments can be found in the data archive for this paper: https://osf.io/xyq4h/). These failures are all the more striking given that each experiment added detail intended to give subjects every chance to evaluate the knots accurately (including variations specifically inspired by critiques of previous intuitive physics tasks), and also employed catch trials that all included subjects answered correctly. In other words, subjects understood their task, and demonstrated that they were capable of making at least some minimal evaluation of knot strength (albeit in a fairly trivial case). These results thus continue to suggest that knots pose a particular challenge to human physical reasoning.

## EXPERIMENT 5: KNOT IDENTIFICATION VS. KNOT EVALUATION

Experiments 1–4 provide evidence for striking failures in knot strength evaluation, across many variations in presentation. However, it may still be that these results do not reflect failures of physical understanding per se, but rather a more general failure of visual cognition to extract the topology of the knots from the presented images. In other words, perhaps errors reflect impoverished *inputs* to the physical reasoning mechanism, rather than the operation of the physical reasoning mechanism itself. This may be true even for Experiment 4, which presented schematic diagrams alongside the knots; though our intention was that this additional information would facilitate extraction of topology (and thereby enable accurate strength judgments), perhaps these schematics simply failed to achieve this goal.

As a check on this possibility, Experiment 5 employed a similar design as Experiment 4, but instead of making strength judgments, participants simply matched the knot photographs to their corresponding schematic diagrams. Success in this task is contingent on accurately representing the knots’ topologies; so, if subjects can perform well at this task, then failures in early experiments are unlikely to reflect mere input constraints and instead likely to reflect deeper errors in physical scene understanding.

### Method

This experiment used the same knot photos from Experiments 1 and 4, and the same knot diagrams from Experiment 4. However, in the present task, participants simply matched a photograph of a knot with its schematic diagram. On each trial, a single knot photograph appeared, and beneath it were each of the four schematic diagrams (reef, thief, granny, and grief). Participants clicked on the schematic diagram that they believed represented the knot.

To ensure that the task was clear, participants had to complete four practice trials before they could proceed to the full experiment, where they matched different versions of each knot in a colorway not shown during the full experiment. In the full experiment, each knot (including front and back views) was displayed twice across the same three colorways used earlier, for 48 test trials. In addition to these test trials, randomly during the experiment participants also completed two catch trials where, instead of a knot photograph appearing, a schematic diagram itself appeared, such that one of the four options was just a copy of the central image; this was to ensure that participants were looking at each diagram closely.

### Results and Discussion

**Figure 5.**
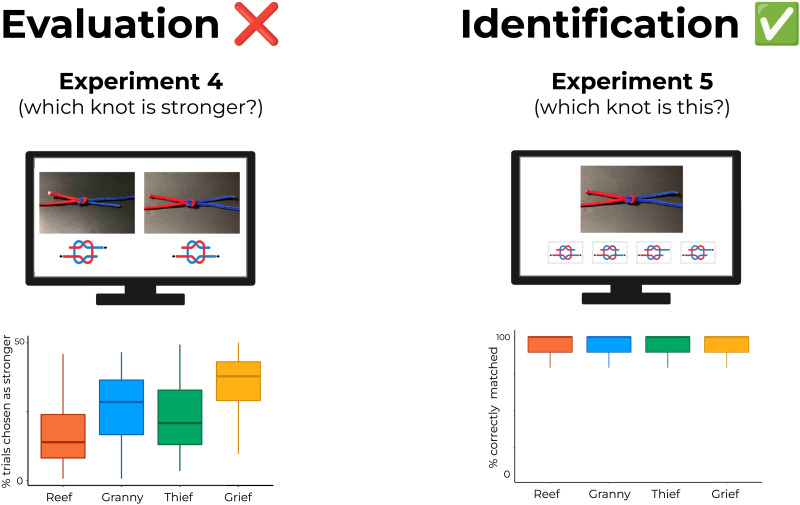
**Results of Experiment 5**. Whereas evaluations (left; Experiment 4) of knot strength showed striking inaccuracies (failing to match ground-truth physics), knot identification (right; Experiment 5) showed striking *accuracy*, with performance near ceiling. In other words, participants *were* able to tell what kind of knot they were viewing (where such discriminations require parsing finer details of the knots); they were just unable to translate that understanding into accurate evaluations of knot strength – in line with our hypothesis that knots are challenging to reason about physically (even when participants can accurately represent their underlying topology).

In principle, this task might have set up participants for worse performance than previous experiments, since the odds of a correct guess on any trial was 1 in 4 rather than 1 in 2. However, performance in this task was exceptional, and indeed even close to ceiling: 92.5% (where chance is 25%), *t*(78) = 44.34, *p* < 0.0001; *d* = 4.99. And even this high average perhaps undersells participants’ performance, due to the skewness of this measure; for example, 68% of participants scored above 95%. Results are shown in Figure 5.

This result suggests that observers can extract the topological properties of these knots after all – or, at least, those details that distinguish the knots from one another. And so the failure to do so is unlikely to be the explanation of poor performance in Experiments 1–4. Put differently: Participants *were* able to grasp the topological properties of the knots; what they were unable to do was derive from that understanding an accurate sense of the physics that such topology entails. Of course, participants were not literally perfect; but occasional errors are not a sufficient explanation of the results of Experiments 1–4. The strongest remaining explanation, then, is that human physical reasoning truly is strained by knot-like stimuli.

## GENERAL DISCUSSION

Whereas recent work documents surprisingly accurate intuitions about a variety of physical phenomena – and uses these successes to posit a general-purpose physical reasoning mechanism – here we have explored a new class of visual stimuli and phenomena that strains physical understanding. Across four experiments, human observers failed to discern even very large differences in the strength of simple knots. Importantly, the errors observed here persisted despite several additions and modifications to the stimuli and task intended to draw out the knots’ mechanical properties. These variations include: Naturalistic photographs (Experiment 1), digital renders from physically precise simulations (Experiment 2), dynamic videos (Experiment 3), and schematic diagrams (Experiment 4). Additionally, these failures were not simply due to an inability to visually extract the topological structure of the knots, since performance was near ceiling in a task that required matching photographs of the knots to their respective schematic diagrams (Experiment 5). In other words, participants were able to discern the structural and topological properties of the knots; what they failed to understand was how this structure translates into corresponding physical and mechanical properties. Moreover, participants were not merely guessing randomly in making their judgments, since many experiments revealed systematic patterns in responding (just not patterns that tracked with the actual strength of the knots). Overall, then, these experiments provide evidence that knots pose a challenge to physical reasoning; and by extension, they place constraints on theorizing about physical scene understanding and the mechanisms underlying it.

It is worth being clearer about the nature and significance of these constraints; what implications do these results have for broader theorizing about general-purpose physical reasoning mechanisms? Though there can, in principle, be many general-purpose accounts of physical reasoning, one especially popular theory in recent years is the Intuitive Physics Engine (IPE) hypothesis (for a review, see Ullman et al., 2017; for an earlier presentation of the core idea, see Battaglia et al., 2013). This account extrapolates from success in certain domains of physical reasoning – such as judging the stability of a tower of blocks, the behavior of connected gears and pulleys, or the flow of a liquid around obstacles (as in Figure 2A and 2B) – to a general-purpose physical simulation device in the mind. This hypothesized device models the physics of the world and the objects within it according to Newtonian laws and principles, with terms for mass, gravity, friction, and other relevant physical parameters; performance on a given physical reasoning task is thus thought to reflect the output of this device and its simulations. Although the IPE is hypothesized to be “noisy” and probabilistic – only approximating scenes and their physics, subject to uncertainty (Battaglia et al., 2013; Sanborn et al., 2013) – it is nevertheless thought to be sufficient for most commonsense visual judgments.

Though our interests here go beyond any particular instance or variation of this hypothesis, the IPE is a useful vehicle for understanding how domain-general physical reasoning might be carried out by the mind – and so is correspondingly useful for thinking through the implications of the present results.

If physical reasoning indeed reflects a domain-general process that models the world according to principles of Newtonian mechanics, then a natural question arises as to why participants consistently failed to appreciate the strength of knots in our tasks. Under the IPE hypothesis, for example, failures in physical reasoning are typically thought to emerge when the stimulus is impoverished or presented without sufficient context (e.g., line diagrams rather than naturalistic images or videos), or the task or physical scenario is unnatural or unfamiliar (e.g., tracing the trajectory of an object exiting a spiral tube; Battaglia et al., 2013; Kubricht et al., 2017). While these factors certainly seem relevant for explaining poor performance in other intuitive physics tasks, it is not clear that they straightforwardly account for the failures we observe here in Experiments 1–4. The stimuli used in our experiments were shown in a variety of presentations designed to maximize both visual context and realism, and Experiment 5 revealed that participants could correctly parse the layout of each knot based on static images. This indicates that the stimuli themselves contained the information that governs differences in their strength, and that participants could access that information in other contexts. What they failed to do, consistently, was translate that information into accurate knowledge of knot strength.

### The Role of Familiarity and Experience

A more open question, perhaps, is how ‘familiar’ or ‘natural’ knots are as a stimulus class, and indeed whether one should expect a domain-general physical reasoning mechanism (whether the IPE or any other mechanism) to apply to them in the first place.

One concern along these lines is that knots may just seem like an overly specialized domain — a skill of interest to sailors and rock climbers but not ordinary people. However, as discussed previously, knots are actually quite pervasive, certainly in contemporary life (tying shoes, untying tangled headphone cords, etc.), across cultures and time periods (where they have been used for millennia for practical, ritualistic, and decorative purposes; d’Errico et al., 2018; Leroi-Gourhan, 1982; Turner & van de Griend, 1996), and even in the practices of other species (Hardy et al., 2020; Herzfeld & Lestel, 2005). Though it is admittedly unclear just how familiar a stimulus must be in order to fall within the purview of a given physical reasoning mechanism (at least under current frameworks), we note that knots seem no *less* familiar than other stimuli that elicit accurate physical intuitions. For example, previous work has shown that naive subjects succeed at tasks that require them to anticipate the behavior of interlocking gears or systems of connected pulleys (Hegarty, 2004). It strikes us that, if naive subjects succeed at *those* (rather unfamiliar) tasks, then unfamiliarity per se may not be a reason to predict failure on knots. (Ask yourself: When was the last time you hoisted an object using a system of interconnected pulleys? And when was the last time you tied your shoes?) And even if our participants were unfamiliar with the specific knots used in our task, these knots are actually *less* complicated than the already rather simple shoelace knot (which in fact contains the reef knot studied in our experiments).

Another way in which knots may be distinct from other kinds of physical stimuli we encounter is that they often represent a form of “received wisdom”; some considerable portion of any individual’s knowledge about knots often comes from instruction, beyond what they may learn from intuitive self-discovery or observation in nature. This aspect of knots raises questions both about the bounds of physical reasoning as well as the role of experience in parsing knots and evaluating their strength. For example, it is quite plausible that expert sailors or rock-climbers might succeed where our naive participants failed, owing to their expertise in recognizing and evaluating knots. However, from our perspective this observation only *strengthens* the implications our results have for theories of intuitive physical reasoning. The fact (if it is a fact) that expertise is required to correctly evaluate the strength of knots and tangles only further testifies to their counterintuitive nature; by contrast, no similar training or expertise seems needed to predict the behavior of interlocking gears or the path of a flowing liquid around various barriers (Bates et al., 2019; Hegarty, 2004). This suggests all the more that knots do not belong to the same class of phenomena that humans can readily and accurately reason about — in line with our interest in them as a case study of everyday physical phenomena that fall outside the scope of domain-general physical reasoning capacities. To put the point another way: While expertise would surely be required to reason correctly about electromagnetism or quantum physics, knots are decidedly *unlike* those systems: Knots are *not* somehow more complicated or obscure than many of the physical stimuli and systems that have been shown to elicit successful reasoning, and yet they nevertheless strain our physical intuitions.

### Rigid-Body Physics vs. Soft-Body Physics

Another possibility underlying failure in this task is that domain-general physical reasoning may be optimized for (or restricted to) rigid-body objects, and that physical reasoning is strained when making predictions about the kinds of soft, flexible materials knots are typically composed of. For example, if human physical reasoning works similarly to a physics engine – perhaps one that prioritizes speed and generality over precision and accuracy – then one might predict difficulties with soft-body objects, as simulating their physical properties is thought to be more computationally demanding than simulating the behavior of simpler geometric rigid-body objects such as stacks of blocks (Ullman et al., 2017). Indeed, realistically simulating knots and ropes has long been a challenge in computer graphics (including in the gaming industry), with various computational techniques developed to approximate different properties. For example, Jakobsen (2001) describes a method in which rope can be simulated in a simple 2D environment by creating a set of particles whose positions are updated to mimic deformations due to gravity and tension, and Phillips et al. (2002) introduce an alternative method where ropes are instead represented as splines of linear springs, and knots can be formed in 3D space by tracking collisions of the rope with itself. A particularly detailed simulator developed by Brown et al. (2004) allows users to manipulate rope in real time and construct knots by modeling rope instead as a cylinder that deforms and stretches over physically motivated constraints. Each of these simulation approaches trades off some degree of realism and accuracy for speed or computational efficiency; it is possible that similar tradeoffs arise in human physical reasoning (perhaps depending on the particular task at hand). That said, it seems unlikely that poor performance in our task could be solely attributed to the non-rigid nature of our stimuli, if only because observers have been shown to make rather accurate predictions and judgments about other non-rigid or soft-body stimuli. Such cases include cloth draped over an object (Wong et al., 2023; Yildirim et al., 2024), liquid pouring into containers (Bates et al., 2019; Kubricht et al., 2016) and elastic objects (Paulun & Fleming, 2020; see also Little & Firestone, 2021). Under current models, it is unclear why observers succeed in these contexts yet fail when asked to judge relative differences in strength between knots. Further research adopting the game-engine approach might shed light on the specific computational constraints of simulation in physical reasoning in a way that accounts for failure to judge the strength of knots while preserving success in other tasks involving soft, flexible materials.

### Heterogeneity in Physical Reasoning

If the above explanations are insufficient, then why did our subjects fail? One possibility is that physical reasoning mechanisms are simply more heterogeneous than a pure simulation-based account would imply, and that the mind employs different physical reasoning strategies depending on stimuli and task demands (see, e.g., Smith et al., 2023). It could even be the case that knots and tangles belong to a special class of objects or systems that cannot be processed by a domain-general physical reasoning mechanism. On this interpretation, when simulation fails (due to computational complexity, resource constraints, or other reasons), subjects may be using heuristics to evaluate the strength of knots, and these heuristics may simply fail to track with knot strength (in at least the present scenarios). Importantly, heuristics may account for the patterns of responses here even though the knots most favored by subjects varied by experiment. For example, if the heuristics subjects used were based (even in part) on some factor that was not systematically varied or measured across experiments — such as, e.g., how tightly wound a knot appeared, whether there was a visible gap between different segments of the knot, or even more incidental factors such as how it rested on the surface where it was photographed — then responses that seem unsystematic *with respect to knot type* could still arise from heuristic reasoning. An open question remains as to just how much of physical reasoning is captured by one or the other approach (simulation vs. heuristics) – an issue raised by recent critiques of the hypothesis that general-purpose simulation is the primary driver of physical predictions (e.g., Ludwin-Peery et al., 2021; Marcus & Davis, 2013; though see Bass et al., 2021). Our work here is agnostic about these broader challenges, though it is certainly possible to see the present failures in this more skeptical light.

Beyond considerations about the class of stimuli knots may or may not belong to, it is also possible that the type of physical judgment used in this task may be beyond the scope of intuitive physical reasoning. While we may quickly and accurately make judgments about properties such as weight, center of mass and projectile motion, perhaps judgments about strength (or at least how much pressure a knot can withstand without capsizing) recruit separate reasoning mechanisms. It has already been demonstrated that, even within the same class of stimuli, physical judgments can converge with or diverge from Newtonian predictions. For example, while participants fail to correctly draw the trajectory of a ball on a pendulum once the string has been cut, they can correctly guess its landing location (Smith et al., 2018). This result has been taken to suggest that prediction and explanation of physical scenes may rely on separate mechanisms; the former reflective of a veridical domain-general model and the latter heavily biased and prone to error.

**Figure 6.**
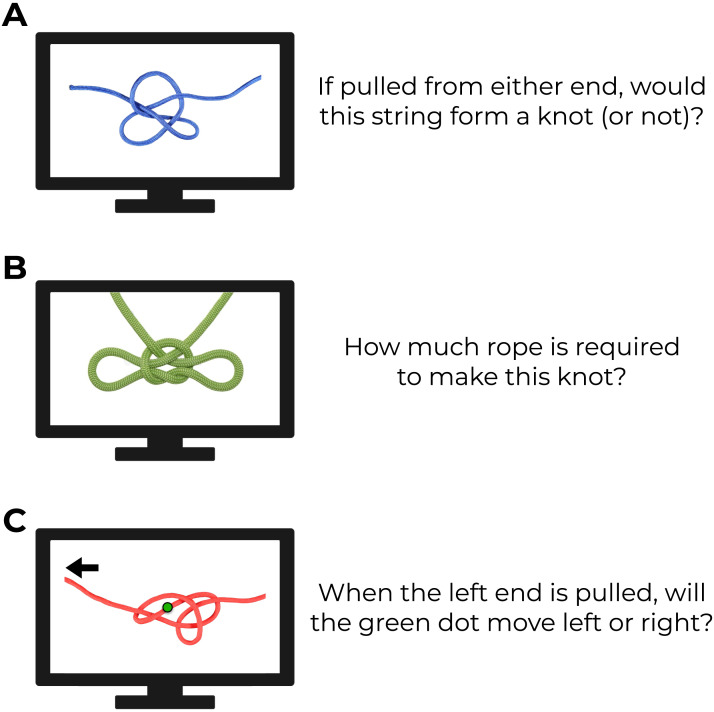
**Other tasks exploring intuitive judgments of knots.** **(a)** How easily can naive participants tell when a tangle of string will form a knot. **(b)** Can we ‘mentally unravel’ bound knots to determine how much string was used to make them? **(c)** A future set of experiments could ask about following elements of a knot as it is loosened or tightened (cf. Hegarty, 2004).

These experiments also open the door to further questions about how people represent and reason about knots. Outside of the challenge they pose to general-purpose theories of physical intuitions, knots have often been seen as having significant (but mostly unrealized) promise to explore physical reasoning more broadly (Santos et al., 2019). For example, even though subjects in our studies struggled to evaluate knot strength, it seems likely that this ability could be acquired through practice and study (and may be present in knot “experts” such as scouts or sailors). In that case, knots could serve as a testbed for physics “training” – the ability to acquire new physical knowledge that is initially unintuitive. There may also be other knot-related tasks that are easier (or harder) for subjects, such as evaluating whether a given configuration of string would or would not become a knot when pulled taut, or even simply estimating how much string is required to make a given knot (see Figure 6).

## CONCLUSION

Physical judgments about the environment are often reliable and robust; but the breadth and depth of physical knowledge may still be both under-examined and under-specified. While relatively unexplored in the domain of intuitive physics, knots provide useful insight into the nature of physical scene understanding – posing a challenge both to reasoners about knots and perhaps even to theories of physical reasoning.

## SIGNIFICANCE STATEMENT

Intuitive physics research has largely focused on rigid-body objects and systems, with recent work revealing strikingly successful reasoning about their physical behavior. The present study introduces a novel stimulus class to this domain of research: knots. Despite being pervasive in everyday life, from tying our shoes to rock climbing, little is known about how well intuitions about the physical properties of knots, such as their resistance to pulling force, map onto their known physical properties. Remarkably, 5 experiments demonstrate that observers fail to produce correct judgments about the strength of very simple knots, revealing a blindspot in theories of physical reasoning. This work may not only prompt further exploration of knots in intuitive physics research (and beyond), but also testifies to the importance of ordinary everyday phenomena that are often overlooked when studying psychological processes.

## ACKNOWLEDGMENTS

For sharing code and resources, we thank the authors of Patil et al. (2020).

## FUNDING INFORMATION

This work was funded by NSF BCS #2021053 awarded to C.F., and NSF Graduate Research Fellowship awarded to S.C.

## AUTHOR CONTRIBUTIONS

S.C.: Conceptualization; Data curation; Formal analysis; Methodology; Writing – original draft; Writing – review & editing. C.F.: Conceptualization; Data curation; Formal analysis; Methodology; Writing – original draft; Writing – review & editing.

## DATA AVAILABILITY STATEMENT

All data, materials, experiment code, and analysis files are available at https://osf.io/xyq4h/. Readers can also visit a project page at https://perceptionresearch.org/knots.

## Figures and Tables

**Figure 7.**
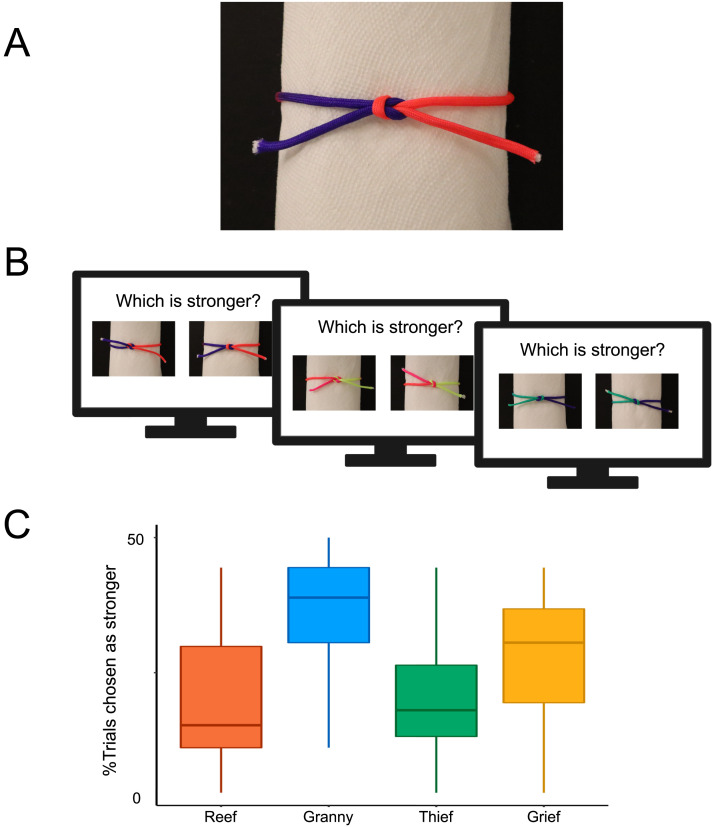
**Experiment 6.** **(a)** A sample binding knot used as stimuli. **(b)**. Each monitor shows a sample trial of Experiment 6, which presents two knots on each trial. Participants simply answered which was stronger. **(c)** Each boxplot represents the distribution of frequencies a knot was chosen as stronger by participants.

## APPENDIX A

One potential concern with the design of our studies is the use of “reef”, “thief”, “granny” and “grief” knots as *bends* (knots that join two pieces of thread) rather than binding knots (a knot made of just one thread, tied to itself, that may be used to keep a single object or multiple loose objects securely fastened, see Figure 7A). As discussed in our main text, the topological mechanics of this knot series has been validated by both optomechanical experiments and computer simulations (see Patil et al., 2020) even when they are used as bends. However, received wisdom from communities that use these knots (e.g., sailors, rock climbers) sometimes holds that even the strongest of these knots is too weak to justify most practical uses. For example, Clifford Ashley, author of an important manual discussed in our text, goes as far to claim that the misuse of reef knots as bends has caused “more death and injury than all other knots combined” (Ashley, 1944, p. 18). The scenarios he has in mind are likely cases where someone has used a reef knot to secure a boat to a dock or to hoist a heavy object into the air.

Despite not being recommended for such sensitive and high-stakes uses, we chose bends for our physical reasoning experiments because both conceptualizing and evaluating their strength is relatively simple (one only needs to consider the pulling forces as well as the implied friction from the strings once tied around each other) and because their strength has been validated in previous work (Patil et al., 2020 – which, again, assesses these knots as bends). Finally, we thought that bends better lent themselves to motor simulation processes, since the task given to subjects is to predict what would happen if they physically pulled on the loose ends of each knot (see Schwartz & Black, 1999, and also discussion in Fischer & Mahon, 2022, who propose that “first-person” or user-oriented tasks produce better physical judgments than third-person problem solving). By contrast, the force applied to binding knots comes from the bound object, rather than a pulling force of the sort that a person could apply.

However, to be sure that the results of our main experiments aren’t due to their presentation as bends, we re-ran Experiment 1 with images of the four knots used as binding knots instead. As noted above, binding knots are typically used to fasten objects; a common maritime application, for example, is to keep a sheet of sail rolled up tightly. Importantly, the communities that rely on these knots still consider the same hierarchy to apply (with *reef* > *granny* > *thief* > *grief*). However, to our knowledge this hierarchy has not been physically validated in the same way as it has for bends (Patil et al., 2020). We thus include this experiment only in this Appendix, because it lacks the kind of ground-truth baseline available for the experiments included in our main text.

### Method

#### Participants.

50 participants were recruited online using Prolific. Each participant was compensated monetarily for their participation. One participant was removed from analysis due to a server error in recording their data. None of the participants failed any of the catch trials.

#### Procedure.

This experiment used the same procedure as Experiments 1–4: A two-alternative forced-choice task between members of the RTGG knot series evaluated for strength. Rather than instructing participants to imagine pulling on either end of the knot, participants were instead asked to infer which knot would be “least likely to let the paper towels unravel”, or “more likely to keep the paper towel bound up”.

#### Stimuli.

The same four knots from Experiments 1–5 were depicted as binding knots instead of bends. For this stimulus set we used 4 mm nylon rope that was tie-dyed so that participants could easily parse how the rope overlapped with itself. Each knot was pulled roughly taut around a bundle of paper towel lying on a black surface, and tied to maximize visual similarity using the length of the bitter ends (the section of a rope that is tied off) as a reference. There were three separate tie-dye colorways (pink/yellow, purple/pink, and green/purple) for each knot, resulting in 12 total images.

### Results and Discussion

This experiment, despite depicting the knots as binding knots instead of bends, yielded very similar results to Experiment 1 (Figure 7C). Overall performance was 46.8% which was not significantly different from chance, *t*(48) = 1.53, *p* = 0.132; *d* = 0.219. This result suggests that the failure to intuit the strength of these knots, as extensively explored and documented in our main text, generalizes to other presentations and does not depend on their depiction as bends.
